# Bacteriophages in Biological Wastewater Treatment Systems: Occurrence, Characterization, and Function

**DOI:** 10.3389/fmicb.2021.730071

**Published:** 2021-09-30

**Authors:** Viviane Runa, Jannis Wenk, Simon Bengtsson, Brian V. Jones, Ana B. Lanham

**Affiliations:** ^1^ Centre for Sustainable and Circular Technologies, University of Bath, Bath, United Kingdom; ^2^ Department of Chemical Engineering, University of Bath, Bath, United Kingdom; ^3^ Water Innovation and Research Centre, University of Bath, Bath, United Kingdom; ^4^ Promiko AB, Lomma, Sweden; ^5^ Department of Biology and Biochemistry, University of Bath, Bath, United Kingdom

**Keywords:** bacteriophages, microbial ecology, wastewater treatment, microbial communities, viruses

## Abstract

Phage bacteria interactions can affect structure, dynamics, and function of microbial communities. In the context of biological wastewater treatment (BWT), the presence of phages can alter the efficiency of the treatment process and influence the quality of the treated effluent. The active role of phages in BWT has been demonstrated, but many questions remain unanswered regarding the diversity of phages in these engineered environments, the dynamics of infection, the determination of bacterial hosts, and the impact of their activity in full-scale processes. A deeper understanding of the phage ecology in BWT can lead the improvement of process monitoring and control, promote higher influent quality, and potentiate the use of phages as biocontrol agents. In this review, we highlight suitable methods for studying phages in wastewater adapted from other research fields, provide a critical overview on the current state of knowledge on the effect of phages on structure and function of BWT bacterial communities, and highlight gaps, opportunities, and priority questions to be addressed in future research.

## Introduction

Bacteriophages, or phages, i.e., viruses that infect prokaryotic organisms, such as bacteria, play an important role in the ecology and evolution of microbial communities. Phage interaction with prokaryotes can influence the composition (Weinbauer and Rassoulzadegan, 2004; Suttle, 2007), function (Thingstad, 2000; Calero-Cáceres et al., 2019), and evolution (Poullain et al., 2008; Koskella and Brockhurst, 2014) of a microbiome. This interaction has been exploited to control microbial growth in environmental, engineered, and medical fields (Nakai and Park, 2002; Lin et al., 2017; Svircev et al., 2018).

Phages are approximately 1 to 2 orders of magnitude smaller than bacterial cells, commonly ranging in size from 20nm to 200nm, with giant phages measuring up to 600nm (Holt et al., 1994; Iyer et al., 2021). They have a simple structure, consisting of a protein capsid containing the phage genome, either single- or double-stranded DNA or RNA (Sharp, 2001; Hatfull and Hendrix, 2011), sometimes with a lipid membrane within the capsid surrounding the genetic material (Lundstrom et al., 1979; Kivelä et al., 2002). Phages usually present a lytic infection cycle that starts with the phage adhering to the bacterial cell wall and injecting its genome into the host. A lytic infection leads to the synthesis of new progeny virions, i.e., infective viral particles, which are released upon lysis of the host cell. However, the phage genome might be incorporated into the bacterial genome without causing lysis of the host. This infection is named lysogenic and expression and releasing of virions only occurs if the host is subjected to an external stress (Adams, 1959; Weinbauer, 2004). Depending on the host’s growing and environmental conditions, life cycles variations can be observed (Duerkop et al., 2012; Howard-Varona et al., 2017; Ogilvie and Jones, 2017).

Engineered biological systems are central to many environmental applications, including treatment of wastewater and production of bioenergy carriers. Wastewater treatment relies on engineered microbiological processes for biological carbon and nutrient removal, such as suspended growth processes (e.g., activated sludge), attached growth and biofilm processes (e.g., trickling filters, moving bed biofilm reactors, and aerobic granular sludge), and anaerobic digesters, defined here as biological wastewater treatment systems (BWT; Henze et al., 2008).

The role of phages in BWT has been of increasing interest to assess phage impact on bacterial consortia and consequently process efficiency and effluent quality (Withey et al., 2005; Shapiro et al., 2010; Wu et al., 2017). However, it has not been studied to the same extent as for other ecosystems, such as marine (Breitbart et al., 2018) and freshwater environments (Palermo et al., 2019), soil (Pratama and van Elsas, 2018), and the human body (Ogilvie and Jones, 2017), particularly in health-related contexts (Lawrence et al., 2019; Principi et al., 2019). More knowledge on the identity, abundance, and role of phages in BWT may allow more robust and efficient operation, one of the key challenges to achieve target levels of wastewater treatment and potentiate water reuse.

This work focuses on phages in BWT and their interaction with microbial communities central to wastewater treatment. Enteroviruses and pathogen indicators are not included in this review and are reviewed elsewhere (Fong and Lipp, 2005; Farkas et al., 2020). To encourage future research on BWT phages, this review discusses methods based on best practice from phage research in other environments (“Methods to Study Phages in BWT Systems”). We then review available knowledge on phage ecology in BWT (“Phages Identified in BWT Processes”) and identify the gaps and opportunities that should lead future research (“Challenges and Opportunities for Studying Phages in BWT Systems”).

## Methods to Study Phages in BWT Systems

A BWT grab sample can be directly used for analysis of phages. However, usually phages are separated from bacteria and other suspended solids by a series of recovery and pre-treatments to obtain a phage suspension. After phage recovery, multiple methods can be used for identification, quantification, and characterization of their activity, as summarized in Table 1. As there is no single methodological pipeline for the isolation and characterization of phages, we propose the most suitable methods to be applied to BWT samples.

**Table 1:** Techniques applied for the study of bacteriophages recovered from environmental samples, highlighting the type and quality of information yielded by each technique, with **+** meaning yes or positive and **–** meaning no or negative. Examples of the applications of the techniques in biological wastewater treatment studies are presented and discussed in section 3.

Technique	Sample requisites			Information Obtained							
	High titer	Genome sequence	Cultivable Host	Quantitative^1^	Qualitative^2^	Phage Specific^3^	Phage-host system	Diversity of phages	Diversity of whole community	Genome Sequence	Applied in BWT
**Culture-based**											
Plaque-assays	−	−	+	+	−	+	+	−	−	−	+
Liquid culture lysis	−	−	+	+/−	−	+	+	−	−	−	−
**Culture-independent**											
Whole-genome sequencing	+	−	−	−	+	+	−	−	−	−	+
Virus-like particles library	+	−	−	−	+	+	−	+	−	+	+
Insert metagenomic library	+	−	−	−	+	+	−	−	−	+/−	+
Whole-community sequencing	+	−	−	+/−	+	−	−	+	+	+	+
Single-cell amplified genome	−	+	−	−	+	+	+	−	−	+	−
Digital PCR	−	+	−	+	+	+	+	−	−	−	−
phageFISH	−	+	−	+	−	+	+	+	+	−	−
**Microscopy**											
Epifluorescence Microscopy	−	−	−	+	−	−	−	−	−	−	+
Transmission Electron Microscopy	−	−	−	+	+	−	−	+	−	−	+

1 Quantitative: yields information regarding phage abundance.

2 Qualitative: yields information regarding phage genome, proteome, or phylogeny.

3 Phage specific: if technique can retrieve information on a specific phage species.

### Methods for the Recovery of Phages From BWT Processes

#### Recovery of Phages From Environmental Samples

**Figure 1:**
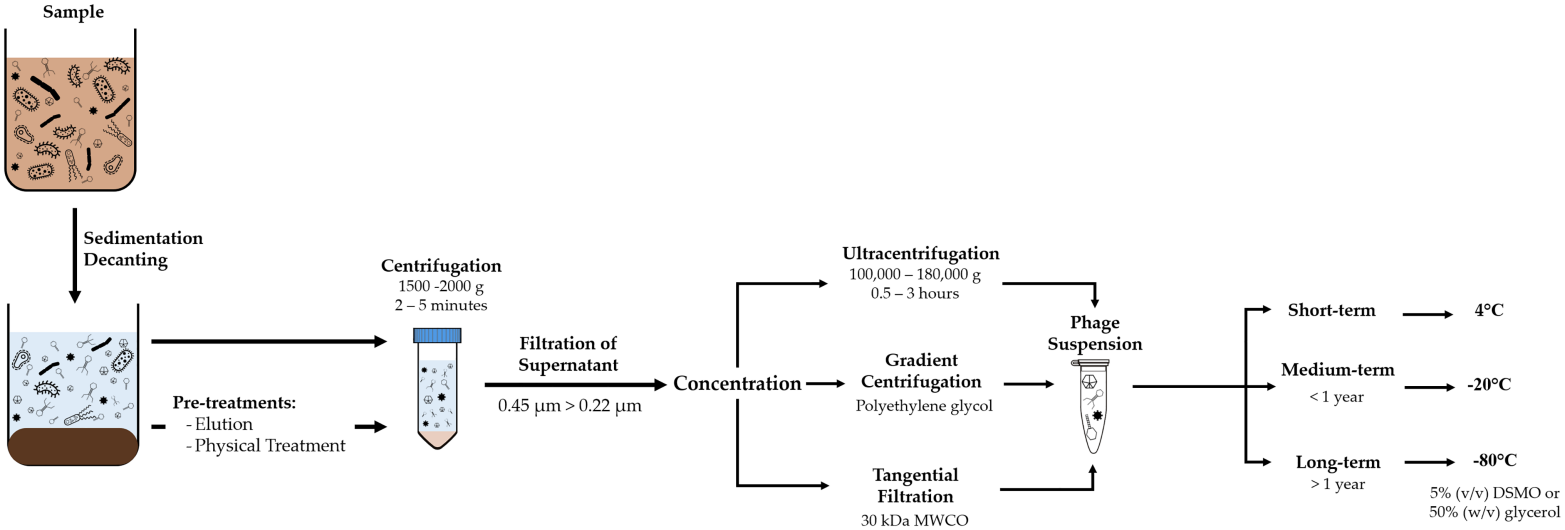
Diagram of methodology for recovery of viruses from environmental samples, comprising removal of suspended solids and concentration of the phage suspension, and adequate storage conditions according to time frame.

Recovery of viruses from environmental samples consists of a series of filtration and centrifugation steps to remove suspended particles, such as sediments, bacteria, and multicellular organisms (Figure 1). The resulting sample should yield a contaminant-reduced viral suspension and can be further purified or concentrated.

Although composition of BWT samples varies depending on the influent and type of treatment, they usually have a high concentration of suspended solids that need to be removed. Processing should include decanting or sieving to remove coarse-suspended solids. Centrifugation between 5,000 and 10,000*g* for 10 to 30min (Shahin et al., 2019) or filtration with polymeric membranes at 0.45μm removes smaller suspended solids and most prokaryotic cells (Logan et al., 1980; Sullivan et al., 2003). A second filtration step at 0.22μm removes remaining bacteria, with possibility of larger phages to be retained as well. For large volume samples or when concentration of the viral suspension is required, tangential flow filtration or gradient centrifugation can be used (Clokie and Kropinski, 2009; Thurber et al., 2009; Kleiner et al., 2015).

#### Pre-treatment Methods for Recovery of Phages From Complex Matrices

Samples with complex matrices, such as activated sludge or anaerobic sludge from BWT, may require additional pre-treatment steps to obtain a phage suspension. Phage adsorption is controlled by the sample’s ionic environment, and therefore, its desorption can be induced by changing the pH and ionic strength (Adams, 1959; Sadeghi et al., 2011). This change can be made by using buffer solutions or surfactants, sometimes combined with physical treatment (Danovaro et al., 2001; Williamson et al., 2003, 2005; Reyes et al., 2010; Hoyles et al., 2014).

Selection of pre-treatment should consider phage distribution across liquid, suspended, and solid phases. Failing to recover phages attached to free bacteria or lodged within flocs, aggregates and biofilms can lead to an underestimation of their quantity and recovery of a non-representative sample (Motlagh et al., 2015; Wu et al., 2017). In fact, it is estimated that over 97% of the phages in activated sludge are adsorbed into suspended solids (Ketratanakul and Ohgaki, 1989). Beef extract solution, potassium citrate, and phosphate solutions have been used as buffers for recovery of viruses from activated sludge from lab-scale systems (Khan et al., 2002a; Motlagh et al., 2015) and from activated and anaerobic sludge samples from full-scale systems (Wu and Liu, 2009). However, beef extract has been associated with interference in downstream analyses of phages, e.g., in fluorescence microscopy (Katayama et al., 2002; Williamson et al., 2003). A combination of physical and chemical pre-treatments can enhance phage recovery (Monpoeho et al., 2001).

To optimize extraction and recovery of phages, more research is needed on how the flocs and granules’ structure hinders phage recovery and what are the prevailing chemical and physical interactions between phages and activated sludge bacteria.

#### Storage of Phage Suspension

Grab samples or recovered phage suspensions should be processed as soon as possible or stored at 4°C to minimize deterioration of phage viability (Clokie and Kropinski, 2009). Phages can be suspended in the working buffer or in 1% (v/v) chloroform to improve sample stability (Mohiuddin and Schellhorn, 2015; Fan et al., 2019). However, chloroform can damage the lipids within the capsid and cause inactivation of some phages and should be avoided when the type of phages is unknown or whole viral communities are to be studied (Clokie and Kropinski, 2009). Samples can be stored at 4°C for short periods of time without significant loss of phage numbers and activity, at −20°C for short/medium term storage, with possible addition of preservative agents, and cryopreserved at −80°C for long term storage with 50% (w/v) glycerol or 5% (v/v) DMSO. However, freezing and freeze-thaw cycles can decrease both phage concentration and activity and introduce bias in subsequent analysis or experiments (Suttle et al., 1991; Thurber et al., 2009).

### Isolation of Phages

Conventional culture-based methods to isolate phage require the growth of host bacterial species as pure cultures, to support the replication of the desired phage. Isolation of a specific phage permits detailed studies of bacteria-virus interactions, infection patterns, and host-phage coevolution (Clokie and Kropinski, 2009; Hyman, 2019).

**Figure 2:**
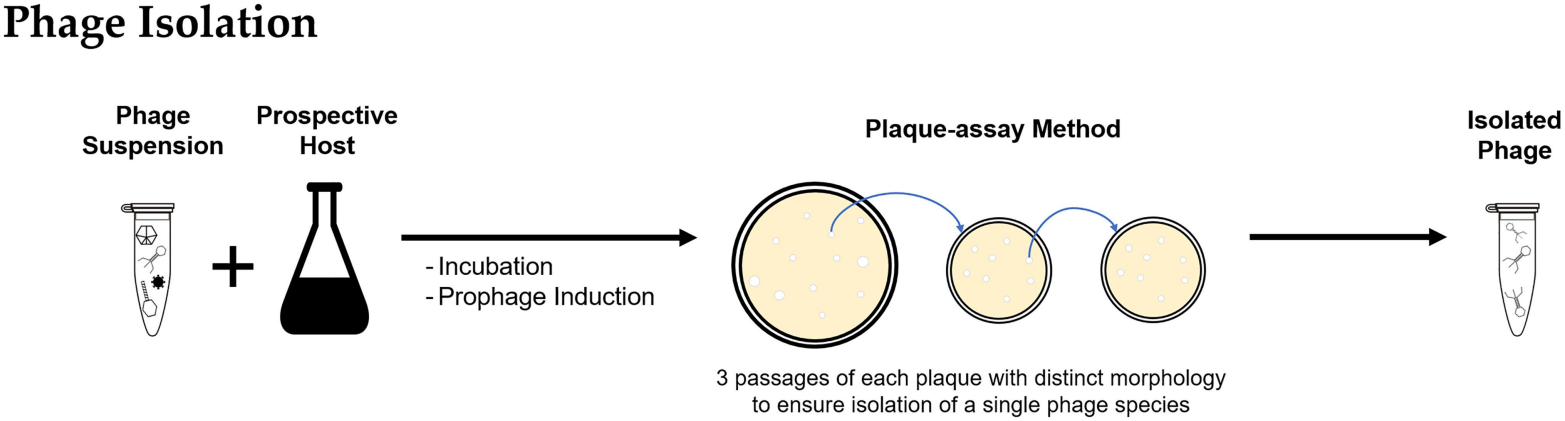
Methodology for bacteriophage isolation: The bacteriophages recovered from environmental samples are incubated with the prospective bacterial host and used to inoculate a plaque-assay; the procedure is repeated using material of a single plaque to ensure isolation of individual phage types.

After recovery and purification, the phage suspension might contain the target phage but likely in low numbers. Suspensions can be incubated with a pure culture of the prospective bacterial host as an enrichment step to promote phage propagation and obtain higher concentrations (Figure 2; Clokie and Kropinski, 2009; Bonilla et al., 2016). The resulting phage suspension can be used as inoculum for a plaque-assay (Adams, 1959; Clokie and Kropinski, 2009; Hyman, 2019) by either directly applying the suspension into a lawn of the bacterial host or by using the double-agar/agar overlay method (Hantula et al., 1991; Lee et al., 2006). For lytic infections, as the phage progeny is released and infects neighboring bacterial cells, a transparent plaque visible to the naked eye is formed. Often plaques have varying morphologies that are likely formed by different types of phages present in the environmental sample. For purification of individual phage types, material from a single plaque is used to infect a fresh host culture, and the enrichment and plaque passage are repeated multiple times until all plaques observed in a plate have the same morphology, to ensure the isolation of a single phage species.

**Figure 3:**
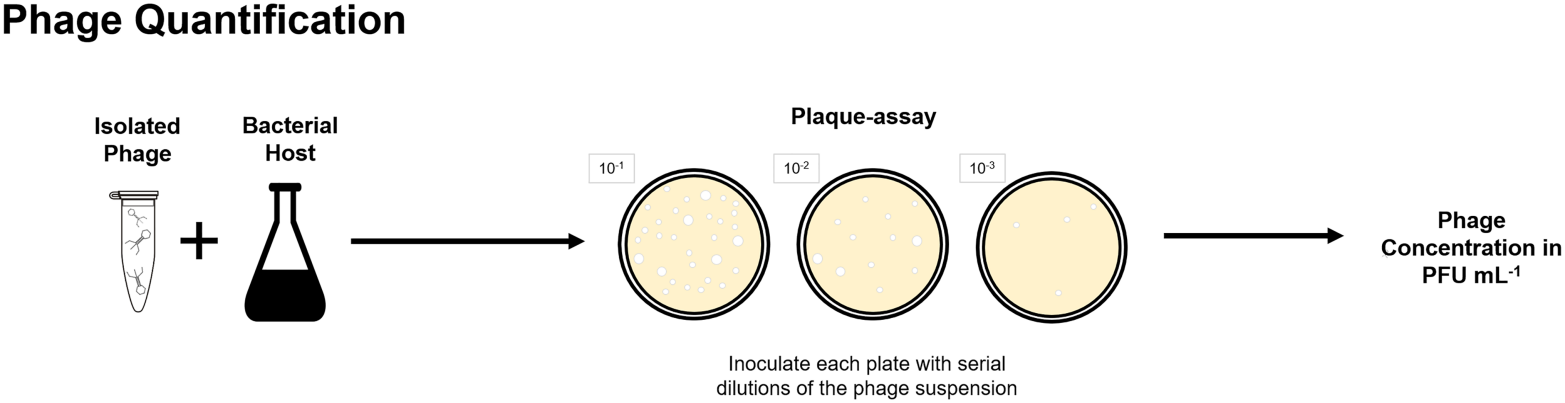
Plaque-assay method can be used to determine the concentration of a phage suspension in plaque forming units per volume, in which each plaque is considered to be originated by one single phage.

Culture-based methods were applied for phage isolation from lab-scale and full-scale enhanced biological phosphorus removal (EBPR) systems (Khan et al., 2002b; Lee et al., 2004, 2006) and full-scale activated sludge plants (Fan et al., 2017, 2019). Further examples on isolation of phages from BWT systems are provided in “Phages Isolated From BWT Systems.” Quantification of isolated phages is routinely done by plaque-assays and determined as plaque forming units per volume (Figure 3) but alternative techniques for phages quantification are described in “Analysis of Phages Genome” and “Uses of Microscopy to Quantify and Characterize Phage Morphology.”

Phages causing a lysogenic infection, also described as temperate phages, can be challenging to investigate through plate-based assays as they do not always readily replicate or produce a plaque and can be difficult to propagate in the enrichment steps. To induce prophage replication and virions release, stress factors, such as antibiotics or DNA compromising agents, including mutagenic compounds, such as mitomycin-C (Choi et al., 2010; Motlagh et al., 2015) and radiation (Clokie and Kropinski, 2009), can be applied to the host cells.

### Analysis of Phages Genome

Genetic analysis of isolated phages can reveal information about phage life cycle and interaction with the bacterial host. Genome sequencing is also valuable information that can be published and help unveil the diversity and role of phages in BWT. Genome information of phages is accessed via genetic sequencing. The first step involves DNA and/or RNA extraction and purification, following established protocols (Green and Sambrook, 2012; Iker et al., 2013; Jakociune and Moodley, 2018). Various sequencing methods, e.g., Illumina sequencing (Rihtman et al., 2016), Ion torrent (Marine et al., 2020), or pyrosequencing (Becker et al., 2012), can be applied for phages following standard procedures developed for bacteria characterization. Bioinformatic tools enable the assembly the sequencing reads and comparison against genome databases, such as GenBank, EMBL, and DDBJ (Hatfull and Hendrix, 2011; Walker et al., 2018). Phage specific databases, such as PhagesDB, are also available (Lima-Mendez et al., 2007; Leplae et al., 2010; Russell and Hatfull, 2017). Some studies on the isolation of phages from BWT carried out genomic analysis, as further discussed in “Phages Isolated From BWT Systems” (Petrovski et al., 2011a,b, 2013, 2014; Khairnar et al., 2014; Liu et al., 2015; Fan et al., 2019).

Obtaining the phage genome is necessary for the identification of functional genes but also enables other analysis. For instance, it permits the assigning of phages to viral families. Viruses taxonomy is an evolving area with an increasing number of phage genomes being added to databases and continuous evaluation of established taxonomic groups (Hatfull and Hendrix, 2011; Tolstoy et al., 2018). Viral taxonomy still has some challenges, such as lack of universally conserved phylogenetic markers in phage genomes (analogous to 16s rDNA in bacteria) or adaptation of available methods to smaller genomes, which consequently deters the unveiling of phage diversity in BWT. Knowing a phage genome can too enable its quantification. If the phage genome is known, fluorescence quantitative polymerase chain reaction using targeted PCR primers tagged with a fluorophore can be used to detect and quantify the phage (Imamovic et al., 2010; Flannery et al., 2013; Kitajima et al., 2014). The limited knowledge on phages found in BWT hinders the design of suitable primers, restricted to the known sequences (Girones et al., 2010; Bibby et al., 2019), and biases during genome amplification, such as uneven coverage of viral genomes, over-amplification of dominant groups, and limitation to dsDNA template, need to be considered for accurate quantification (Kim et al., 2013a; Brinkman et al., 2018). Although genome sequencing and analysis is not fully explored in published studies (Table 2), they represent important opportunities for future phages isolated from BWT systems.

**Table 2:** Lytic phages isolated or identified in BWT systems using culture-based methods.

System	Isolates	Phages	Bacterial Host for isolation	Method	Polyvalence (+/−)	Loss of virulence (+/−)	Ref
Full-scale AS and surroundings^3^	10	AO-1	M1-W91	Double-agar overlay	+	na^1^	Ogata et al., 1980
		AO-2	M1-W83			+		na
		AO-3	M1-W93			−		na
		AI-1	M1-P34			−		na
		BI-1	M1-W14			+		na
		AI-2	M1-W85			+		na
		AIO-1	M1-W95			**−**		na
		AIO-2	M1-W95			**−**		na
		CI-1	M1-W95			**−**		na
		CI-2	M1-P95			**−**		na
Full-scale AS	47^2^	Φ3010	na	Double-agar overlay	**−**	na	Hantula et al., 1991
		Φ3038	na			na		na
		Φ3063	na			+		na
		Φ3064	na			na		na
		Φ3085	na			na		na
		Φ3097	na			+		na
		Φ3122	na			+		na
		Φ4041	na			na		na
		Φ4085	na			na		na
		Φ4086	na			na		na
		Φ4094	na			+		+
		Φ5022	na			na		na
		Φ6004	na			na		na
		Φ6058	na			na		na
		Φ6115	na			na		na
		Φ7039	na			na		na
		Φ7050	na			na		na
		Φ7051	na			na		na
		Φ7066	na			+		na
		Φ7087.1	na			+		na
		Φ7087.2	na			+		na
Lab-scale EBPR^4^	8	ΦP26	*Escherichia coli*	Spot test	+	+	Khan et al., 2002a
		ΦP27	P-27			+		**−**
		ΦP30	*Photobacterium logei*			+	**−**	
		ΦP35	P-35			+	**−**	
		ΦP36	P-36			+	+	
		ΦP37	*Sphingobacterium* spp			+	**−**	
		ΦP38	P-38			+	**−**	
		ΦP40	P-40			+	+	
Lab-scale EBPR	4	ΦL11	L11	Spot test	+	**−**	Khan et al., 2002b
		ΦL15	*Brevibacterium epidermidis*			+		**−**
		ΦL17	*Brevibacterium linens*			+		+
		ΦL22	*Rhodococcus erythropolis*			**−**		+
Full-scale, na	1	HHY-phage	*Haliscomenobacter hydrossis*	Double-agar Layer	**−**	**−**	Kotay et al., 2011
Lab-scale EBPR	40	φE4a	*Caulobacter sp*	Double-agar Layer	**−**	+	Lee et al., 2004
		φE4b	*Caulobacter sp*			**−**		+
		φE8a	*Caulobacter sp*			**−**		+
		φE8b	*Flavobacterium columnare* B2			**−**		**−**
		φE8c	*Flavobacterium columnare* B2			**−**		**−**
		φE8d	*Flavobacterium columnare* B2			**−**		**−**
		φE10a	*Flavobacterium columnare* LP8			**−**		+
		φE10b	*Flavobacterium columnare* LP8			**−**		**−**
		φE10c	*Flavobacterium columnare* LP8			**−**		**−**
		φE10d	*Flavobacterium columnare* LP8			**−**		**−**
		φE10e	*Flavobacterium columnare* LP8			**−**		**−**
		φE10f	*Flavobacterium columnare* LP8			**−**		**−**
		φE10g	*Flavobacterium columnare* LP8			**−**		**−**
		φE10h	*Flavobacterium columnare* LP8			**−**		**−**
		φE10i	*Flavobacterium columnare* LP8			**−**		**−**
		φE10j	*Flavobacterium columnare* LP8			**−**		+
		φE10k	*Flavobacterium columnare* LP8			**−**		**−**
		φE14a	*Bosea* sp			**−**		+
		φE14b	*Bosea* sp			**−**		+
		φE14c	*Bosea* sp			**−**		+
		φE14d	*Bosea* sp			**−**		+
		φE14e	*Bosea* sp			**−**		+
		φE16a	*Pseudoxanthomonas broegberinensis* B1616/1			**−**		+
		φE16b	*Pseudoxanthomonas broegberinensis* B1616/1			**−**		**−**
		φE16c	*Pseudoxanthomonas broegberinensis* B1616/1			**−**		**−**
		φE16d	*Pseudoxanthomonas broegberinensis* B1616/1			**−**		+
		φE16e	*Pseudoxanthomonas broegberinensis* B1616/1			**−**		**−**
		φE16f	*Pseudoxanthomonas broegberinensis* B1616/1			**−**		**−**
		φE16g	*Pseudoxanthomonas broegberinensis* B1616/1			**−**		**−**
		φE16h	*Pseudoxanthomonas broegberinensis* B1616/1			**−**		**−**
		φE16i	*Pseudoxanthomonas broegberinensis* B1616/1			**−**		**−**
		φE16j	*Pseudoxanthomonas broegberinensis* B1616/1			**−**		**−**
		φE16k	*Pseudoxanthomonas broegberinensis* B1616/1			**−**		**−**
		φE16l	*Pseudoxanthomonas broegberinensis* B1616/1			**−**		**−**
		φE16m	*Pseudoxanthomonas broegberinensis* B1616/1			**−**		**−**
		φE16n	*Pseudoxanthomonas broegberinensis* B1616/1			**−**		**−**
		φE16o	*Pseudoxanthomonas broegberinensis* B1616/1			**−**		**−**
		φE16p	*Pseudoxanthomonas broegberinensis* B1616/1			**−**		+
		φE16q	*Pseudoxanthomonas broegberinensis* B1616/1			**−**		**−**
		φE16r	*Pseudoxanthomonas broegberinensis* B1616/1			**−**		+
Lab-scale EBPR	2	ΦMP1	*Microlunatus phosphovorus*	Double-agar Layer	**−**	na	Lee et al., 2006
		ΦMP2	*M. phosphovorus*			**−**		na
Full-scale AS	24	na	na	na	na	na	Parsley et al., 2010
Full-scale AS	1	AJO1	*Acinetobacter johnsonii*	Double-agar Layer	+	na	Fan et al., 2017
Full-scale AS	1	AJO2	*Acinetobacter johnsonii*	Double-agar Layer, Genetic sequencing	+	na	Fan et al., 2019
Full-scale AS	4	GordTnk2	*Gordonia* spp	Spot test, Genetic sequencing	+	na	Liu et al., 2015
		Gmala1	*Gordonia malaquae*			+		na
		GordDuk1	*Gordonia* spp			**−**		na
		Gsput1	*Gordonia sputi*			**−**		na
Full-scale AS	1	TPA2	*Tsukamurella* spp	Spot test	+	**−**	Petrovski et al., 2011b
Full-scale AS	1	GTE2	*Gordonia terrae*	Double-agar overlay	+	na	Petrovski et al., 2011a
Full-scale, na	1	GTE7	*Gordonia terrae*	Double-agar overlay	+	na	Petrovski et al., 2011c
Full-scale, na	3	NOC1	*Nocardia rhodochrous*	Double-agar overlay	**−**	na	Khairnar et al., 2014, 2016
		NOC2	*Nocardia amarae*			+		na
		NOC3	*Nocardia pinensis*			+		na
Full-scale, na	17	GTE1	*Gordonia terrae*	Spot test	+	na	Thomas et al., 2002
		GTE2	*Gordonia terrae*			+		na
		GTE3	*Gordonia terrae*			+		na
		GTE4	*Gordonia terrae*			+		na
		GTE5	*Gordonia terrae*			+		na
		GRU1	*Gordonia rubropertincta*			+		na
		GRU2	*Gordonia rubropertincta*			+		na
		MPH1	*Mycobacterium phlei*			+		na
		NAS1	*Nocardia asteroides*			+		na
		NBR1	*Nocardia brasiliensis*			+		na
		NBR2	*Nocardia brasiliensis*			**−**		na
		NBR3	*Nocardia brasiliensis*			+		na
		RGL1	*Rhodococcus globerulus*			+		na
		RGL2	*Rhodococcus globerulus*			+		na
		RRU1	*Rhodococcus ruber*			+		na
		RER1	*Rhodococcus erythropolis*			+		na
		TPA1	*Tsukamurella paurometabola*			**−**		na

1 na – Not applicable, not specified.

2 Not all isolates were individually specified.

^3^AS: activated sludge. ^4^EBPR: Enhanced Biological Phosphorus Removal.

If phage isolation is not possible or a more comprehensive overview of the phage community is intended, metagenomics can be applied to analyze all nucleic acids recovered from an environmental sample, providing a global assessment of diversity and distribution of phage communities as whole (Angly et al., 2006; Reyes et al., 2013a; Brum and Sullivan, 2015). The application of metagenomics methods starts with the extraction of the total DNA or RNA from either the microbial community or just from the recovered and purified virus particles (Thomas et al., 2012; Culligan et al., 2014). Then, a library is constructed from purified nucleic acids, as reviewed elsewhere (Solonenko and Sullivan, 2013). These are sequenced and aligned with reference databases to identify coding sequences (Kim et al., 2013b; Kelleher et al., 2015). Two different approaches are used within metagenomics to study uncultured organisms. The first employs amplicon sequencing, a targeted technique where primers are used to amplify gene variants of interest from DNA extracts, such as 16S ribosomal RNA genes for prokaryotes. The amplified DNA is then sequenced to obtain coverage of the variants in a population (Pérez-Losada et al., 2020). However, there is no identified genetic marker or conserved sequence for phages and the inability to design primers for phages with unknown sequences compromises an accurate identification of phages and their phylogeny (Angly et al., 2005; Gilbert and Dupont, 2011). Alternatively, shotgun sequencing covers all nucleic acids in a sample (Hayes et al., 2017). Comparative analysis of phage genome constitutes another challenge due to limited availability of viral sequences in databases, and interference of bacterial DNA with viral nucleic acids (Roux et al., 2013; Hayes et al., 2017). Application of these methods in the context of BWT is discussed in “Effects of the Phage Population on Microbial Communities and BWT Performance.”

### Uses of Microscopy to Quantify and Characterize Phage Morphology

Microscopy does not require phage cultivation or isolation and provides information on their morphology and abundance, which can be useful to characterize and infer about phages life cycle and study the population dynamics in a given system.

Provided phage concentration is high enough for sample preparation, morphology and component size can be determined by transmission electron microscopy (TEM), a technique well established, relatively simple to perform, and that reveals unique information over other approaches (Adams, 1959; Ackermann, 2012). TEM has been used to observe morphology of phages isolated from BWT and classify phages into viral families (Lee et al., 2006; Liu et al., 2015; Fan et al., 2017). We encourage the use of TEM to complement the information obtained by genome sequencing and correctly characterize the phages, highlighting that it can also be used to visualize non-cultured phages (Ackermann, 2012; Richert-Pöggeler et al., 2019).

**Figure 4:**
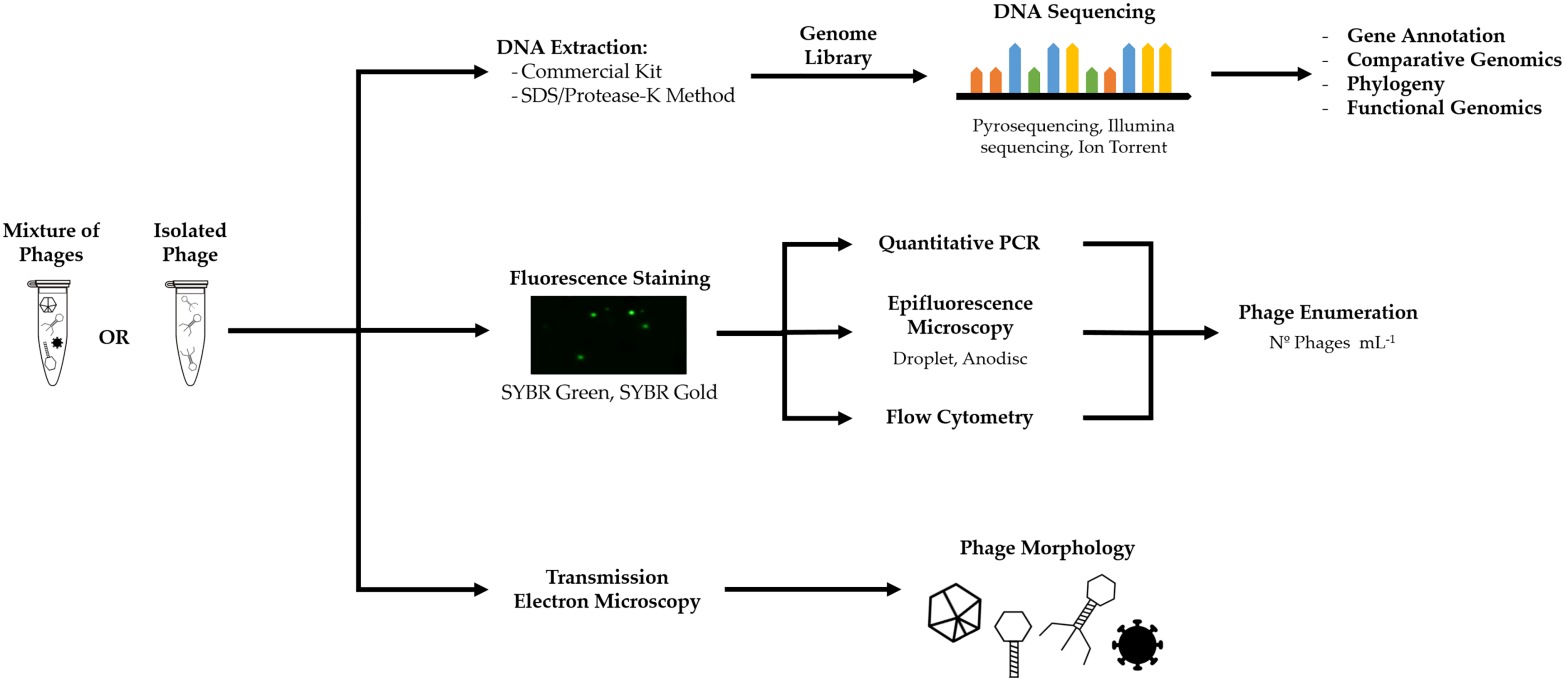
Culture-independent methods, such as metagenomics, fluorescent staining, and electron microscopy, can yield information on phage genome, infection and life cycle, phage concentration, and morphology.

Phages can be quantified via fluorescence-based microscopy or cell counting when stained with a fluorophore (Figure 4; Wen et al., 2004; Pollard, 2012). Common fluorescent stains are SYBR Green I and II ad SYBR Gold (Shibata et al., 2006; Patel et al., 2007). However, staining does not discriminate between phages and other viruses and provides the total number of viral particles in a sample. Occurrence of free DNA can also contribute to an overestimation of phages (Patel et al., 2007; Clokie et al., 2011).

In BWT research, epifluorescence microscopy has been used for the quantification of viruses from full-scale treatment plants (Otawa et al., 2007; Wu and Liu, 2009; Yang et al., 2017) and lab-scale EBPR reactors (Barr et al., 2010; Motlagh et al., 2015). Fluorescence flow cytometry using SYBR Green staining has also been applied for quantification of viruses from activated sludge samples, benchmarked with TEM counts (Brown et al., 2015, 2019). Although other viruses and eukaryotic pathogens can be found (Aw et al., 2014), it is assumed that the majority of viruses in bacteria-rich environments, such as activated sludge, are phages, as these need an active host to propagate and thrive in the system (Payan et al., 2005; Jofre et al., 2014). Nonetheless, it is not possible to make an accurate quantification of all phages in a viral or microbial community.

## Phages Identified in BWT Processes

The presence of phages in activated sludge was first confirmed in 1965 (Dias and Bhat, 1965). Later, activity of phages was correlated to decreasing concentration of its bacterial host in activated sludge (Ogata et al., 1980) while comparison of phage concentration in the influent and along a wastewater treatment plant suggested phage production occurred in the activated sludge tank (Ewert and Paynter, 1980). The average concentration of viral-like particles (VLP) in BWT processes ranges from 10^7^ VLP ml^−1^ to above 10^9^ VLP ml^−1^ which is comparable to the concentration of 10^8^ to 10^9^ VLP *g*^−1^ feces in the human gut (Kim et al., 2011) but two orders of magnitude higher than the values reported for marine environments (Breitbart et al., 2018), groundwater (Pan et al., 2017), and freshwater systems (Filippini and Middelboe, 2007).

Research on phage ecology of BWT has applied both culture-based and culture-independent methods, but many knowledge gaps remain on the abundance and composition of phage community in BWT and factors that can contribute to their interaction with the microbial community, particularly with bacteria performing key functions. Additional studies are necessary to frame more comprehensive insights, with scope for emerging technologies to be used. With increasing evidence of the effect of phages in BWT being reported (discussed in “Effects of the Phage Population on Microbial Communities and BWT Performance”), they may have a more significant role in BWT communities than what has been considered so far.

### Phages Isolated From BWT Systems

**Figure 5:**
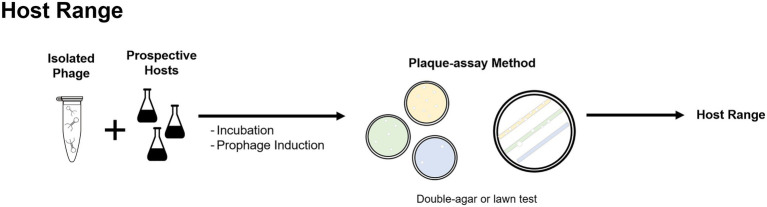
Plaque-assay method can be used to determine the host range of isolated phages by incubating the bacteriophages with selected prospective hosts.

Culture-based methods have been the main technique used to identify phages in BWT systems. In early studies on full-scale activated sludge systems, 10 phages from an aeration tank and surroundings were isolated, using two different bacterial strains isolated from the sludge as prospective hosts (Ogata et al., 1980). A similar screening studied 49 phage-host systems occurring in an aeration basin at a municipal wastewater treatment plant (Hantula et al., 1991). More recently, phages have been isolated from full-scale (Fan et al., 2017, 2019) and lab-scale BWT systems (Khan et al., 2002a,b; Lee et al., 2004, 2006). Different phages infecting foam-formation associated filamentous bacteria from the genera *Dietzia*, *Gordonia*, *Nocardia*, *Rhodococcus*, *Tsukamurella, Haliscomenobacter*, and *Mycobacterium* have been isolated (Thomas et al., 2002; Kotay et al., 2011; Khairnar et al., 2014), sequenced (Petrovski et al., 2011a,b,c, 2012, 2013; Liu et al., 2015), and respective host range studied (Petrovski et al., 2011a,c). Similarly, culture-based methods are the standard in the determination of a phages’ host range, with screening of phages against prospective hosts (Figure 5), and have been used to study the host range of phages isolated from BWT samples (Thomas et al., 2002; Lee et al., 2006; Fan et al., 2019).

As shown in Table 2, studies of phage isolation from BWT systems are still limited. Despite the limitations of culture-based methods, such as loss of virulence (Hantula et al., 1991; Khan et al., 2002a,b) or changes in polyvalency – the ability to infect multiple hosts (Ogata et al., 1980; Thomas et al., 2002; Weinbauer, 2004) – (see “Challenges and Opportunities for Studying Phages in BWT Systems”), the conventional, straight-forward methods for isolation could be more widely applied.

### Effects of the Phage Population on Microbial Communities and BWT Performance

#### Diversity of Phages in BWT Systems

Metagenomics are increasingly used to investigate microbial communities as a whole (Mizuno et al., 2013; Brum and Sullivan, 2015) and have been applied to study the virome diversity in BWT. These culture-independent methods can be used alone or to complement culture-based methods (Figure 4). Although phages are likely the dominant types of viruses in BWT environments (Weitz, 2015), it is not always possible to selectively count or identify phages among the viral population, including enteric or archaeal viruses. Therefore, the term viruses will be used when the methods applied do not enable this distinction. The overall diversity of phages found in wastewater treatment plants is lower that the diversity in marine environments, similar to the diversity, found in soils but higher than the viral diversity in human feces (Tamaki et al., 2012). Abundance and diversity of phages typically change in different ways along the process. The virus load of raw sewage has been estimated at 10^8^ viral particles ml^−1^ (Wu and Liu, 2009; Brown et al., 2019). Concentration of viruses increases in the secondary treatment units to about 10^9^ viral particles ml^−1^ in activated sludge processes (Otawa et al., 2007; Wu and Liu, 2009) and 10^10^ viral particles ml^−1^ in anaerobic digesters (Tamaki et al., 2012), followed by lower concentrations found in the effluent (Otawa et al., 2007; Wu and Liu, 2009). The increase in the concentration of viruses in the treatment course is presumably due to the presence of hosts that promote phage propagation (Hantula et al., 1991). The abundance of viruses decreases in the treated effluent, with a concentration of 10^8^ viral particles ml^−1^ (Wu and Liu, 2009). Although no explanation is provided for the observed decrease, it is likely that viruses are removed along with bacteria in the settling stage. The effluent will furthermore have a negligible concentration of prospective hosts when compared to the previous treatment steps. It can be assumed that virus concentrations will be further reduced if tertiary treatment is applied. Different wastewater treatment plants will likely have variations in phage concentrations depending on influent and operational conditions (Otawa et al., 2007; Wu and Liu, 2009; Petrovich et al., 2019). A few studies have used sequencing methods for virome characterization in BWT systems. Comparative metagenomics of viruses in activated sludge found the viral families *Myoviridae*, *Siphoviridae*, and *Podoviridae*, all within the *Caudovirales* order, at relative abundance of 40.3, 31.9, and 25.6%, respectively (Parsley et al., 2010). Other metagenomics studies on activated sludge (Tamaki et al., 2012; Yang et al., 2017; Petrovich et al., 2019), raw sewage (Aw et al., 2014), and anaerobic digestion (Heyer et al., 2019) also identified these viral families as dominant. However, only about 5% of the sequences obtained in these studies overlapped with known viral genomes in databases, indicating a unique virome in BWT systems. Despite identification of dominant viral families, the virome significantly differs at genera and species level when comparing bulking and non-bulking activated sludge (Yang et al., 2017) or suspended growth (activated sludge) with attached growth (trickling filters; Petrovich et al., 2019).

Similar treatment systems might share a significant part of their phage population including a characteristic genomic fingerprint, i.e., a common or established core of phages characteristic of a treatment process or geographic area, as observed in a study of the viral genome of 14 wastewater treatment plants (Otawa et al., 2007). This is observed for prokaryotic communities of BWT worldwide sharing a common core of organisms (Wu et al., 2019), with differences in some bacterial groups depending on processes and operation mode (Wagner and Loy, 2002; Jo et al., 2016). Furthermore, if metagenomic studies include the analysis of the bacterial genome, fingerprints will then include not only “free” phages but also prophages (Parsley et al., 2010; Petrovich et al., 2019). Establishing a genomic fingerprint can also help addressing questions regarding dynamics in phage-host systems, decline of certain key functional bacteria, and migration of phages within the system.

Metagenomics can produce valuable data which continuously endorses the improvement of genome analysis, expansion of databases, and the identification of patterns in phage abundance and diversity. This information can provide evidence on the uniqueness of BWT phage community and assist with building a useful viral fingerprint for process monitoring and control (Hatfull, 2008), as further discussed in “Challenges and Opportunities for Studying Phages in BWT Systems.”

#### Dynamics of Phage and Bacterial Populations

The type of infectious cycle affects bacterial population dynamics and function differently. Lytic infection can result in sharp decreases in the number of bacterial cells (Thingstad, 2000; Maslov and Sneppen, 2017), while a lysogenic infection proceeds without affecting cell numbers, with the prophage remaining latent within the bacterial genome (Howard-Varona et al., 2017; Monteiro et al., 2019). However, multiple variations of these life cycles can be found in nature.

It has been demonstrated in lab-scale BWT systems that phages can shape microbial communities and process performance. For example, *Microlunatus phosphovorus* concentration in an activated sludge reactor decreased upon phages supplementation (Lee et al., 2007). Sudden failure of a EBPR reactor fed with synthetic wastewater and enriched in *Accumulibacter* was attributed to an infection (Barr et al., 2010). Phosphorus removal of analogous well-performing EBPR systems deteriorated when infected with recovered phages from the original reactor, verifying phage infection over operational conditions or microbial competition as cause of deterioration. In another EBPR system, prophages were induced with chemical stress factors to adopt a lytic cycle, causing a decrease of *Accumulibacter* spp. abundance along with a decrease on phosphate uptake and release (Motlagh et al., 2015).

Similar dynamics between endogenous phage and bacterial populations were observed in full-scale systems. In a membrane bioreactor treating industrial wastewater, phage concentration was inversely correlated with both the original bacterial hosts and phylogenetically related bacteria (Shapiro et al., 2010). An increase of bacteria concentration in a bulking activated sludge was concurrent with a decrease in phages concentration and diversity (Yang et al., 2017). In a nitrification process, a significant correlation between increasing virus number and decreasing total bacteria number was found. The same study showed that virus numbers were significantly influenced by pH and magnesium ions which affect the adsorption of phages to the host (Brown et al., 2019). However, when viruses of 14 different wastewater treatment plants were supplemented to virus-free lab-scale reactors fed with synthetic wastewater (Otawa et al., 2007), phage-host interactions led to relatively slow changes in virus numbers, suggesting a combined effect of lysogenic infections, bacterial resistance, and sludge properties.

Although lytic infections are more easily identified by the steep decrease in host numbers, prophage inclusion in the host genome can benefit the host and indirectly regulate the propagation of other bacteria. For instance, prophage inclusion may grant the host resistance to infection by other phages (Fortier and Sekulovic, 2013), facilitating host growth and pathogenicity (Duerkop et al., 2012; Fortier and Sekulovic, 2013), increasing resistance to multiple stress conditions (Bossi et al., 2003; Wang et al., 2010), and providing advantage in the competition against other bacterial strains (Duerkop et al., 2012; Fortier and Sekulovic, 2013). Rather than causing the decline of the infected population, prophages can instead promote resilience and stability of the bacterial strain in the community, as shown for the gut microbiome (Breitbart et al., 2003, 2008; Kim et al., 2011). It was shown that lytic infections can decrease the production rate of biogas in an anaerobic digester, while facilitating the growth of auxotrophic organisms by increasing nutrient cycling through cell lysis (Heyer et al., 2019). There is also very little information available on how lysogenic activity is regulated in BWT systems, with environmental factors and different pollutants being likely to induce lysogenic cycle (Howard-Varona et al., 2017).

It is still unclear what are the phage-host dynamics in BWT ecology and how significant this impact is on the operation of full-scale communities, e.g., if the affected bacteria would be replaced by other functional organisms or if the development of resistance by the hosts would characterize an arms race of fluctuating-selection model. Without this information, the modeling and control of BWT are lacking a fundamental element.

#### Use of Specifically Isolated Phages for Bacterial Population Control

Lytic phages have potential as biological tools to control bacterial populations. In the context of BWT, phages have been successfully used to control detrimental sludge bulking and foaming in lab-scale systems (Thomas et al., 2002; Withey et al., 2005). Lytic phages have been shown to decrease activated sludge bulking and foaming and to improve process performance by controlling bacteria detrimental to the process (Thomas et al., 2002; Withey et al., 2005). Added phages were able to control foam forming *Haliscomenobacter hydrossis*, without impacting nutrient removal while improving sludge settling properties (Kotay et al., 2011). A mixture of four phages could repeatedly decrease a *Gordonia* population by 90% (Liu et al., 2015). Similarly, addition of three phages infecting *Nocardia* reduced foam production at lab-scale (Khairnar et al., 2016).

Knowledge on the host range is important to design biotechnological applications (Ross et al., 2016; Sharma et al., 2017), as many phages are host specific (Weinbauer, 2004; Poullain et al., 2008) while others can infect a wider range of bacteria across species and genus (Koskella and Meaden, 2013; Ross et al., 2016). Alternatively, a few culture-independent methods are available to study host range and dynamics of phage-host systems, with some techniques developed to study phage infections at a single-cell level, as explained by Dang and Sullivan (2014). Although the techniques described do not require cell cultivation, the information retrieved is restricted by knowledge on the genome of the host and phage of interest and lacks validation and more examples of application (Dang and Sullivan, 2014; Bibby et al., 2019). Even so, they were never applied in the context of BWT and can therefore be an interesting investigation since most bacteria and phages in activated sludge have not been isolated.

Another limitation of the studies on the application of phages as biocontrol agents in BWT is that they all were conducted in lab-scale systems with well-controlled conditions, with microbial communities usually having low diversity. To our knowledge, full-scale testing of phage infection has not been reported but will be necessary for the development of this technology for bacterial population control in BWT. Besides scalability, and as further discussed in “Opportunities and Potential Application of Phages in BWT,” it is also necessary to consider a formulation that ensures activity and effectiveness of the applied phages and how to prevent the development of phage insensitive mutants.

## Challenges and Opportunities for Studying Phages in BWT Systems

### Outlook on Phage Research in BWT

Challenges for studying phages from BWT samples start at the recovery stage. Activated sludge morphology and sample matrix vary depending on the BWT system. Suspended growth technologies, in which bacteria aggregate in flocs, often have higher concentration of suspended solids in the reactor than attached-growth technologies, but generally up to five times lower than in anaerobic digestion systems (Henze et al., 2008). It is still unknown to what extent the physical and chemical properties of activated sludge can compromise an accurate quantification of phages or, more significantly, the success of infection. For instance, granular sludge could be more robust toward phage infection since bacteria performing essential functions in the process aggregate in dense granules and short wastewater residence time promotes the wash out of phages (Danovaro et al., 2001; Brown et al., 2015; Bengtsson et al., 2018). Similarly, biofilms in attached-growth BWT systems can delay phage penetration and infection (Simmons et al., 2020). The effect of phage activity on sludge morphology has also not been explored. Expertise from studies in other environments should be applied to BWT considering the resemblance of sample matrix and composition, e.g., samples from activated sludge systems are more like aqueous or sediment samples while sludge from anaerobic digestion can be processed similarly to soil or fecal samples.

Following to the isolation of phages, culture-based methods have a limited scope of analysis. It is estimated that 99% of all bacteria are yet to be cultivated in lab environments (Rinke et al., 2013). The majority of bacteria from BWT systems has not been isolated (Xia et al., 2018), with many functional organisms only being identified using metagenomic approaches (Nielsen et al., 2019). The impossibility to isolate potential hosts consequently limits the study of phage communities and phages that might be dominant or have pivotal activity in shaping the microbial ecology of a given environment. Even if a prospective host is culturable, other challenges stand before phage isolation. Identifying lysogenic life cycles, which can affect the host populations in different ways (Wang et al., 2010; Reyes et al., 2013b), may require specific media supplementation or culture conditions for induction, which are unknown. Co-evolution of phages and hosts can also lead to the loss of virulence by the phage or development of resistance by the host (Alizon and Van Baalen, 2008; Diard and Hardt, 2017). Bacteria can develop a wide range of mechanisms to avert phage infection at different stages of the process, for instance to inhibit adsorption of the phage to host receptors and the synthesis of intracellular proteins to impede phage genome transcription and maturation (Labrie et al., 2010).

Culture-independent approaches, such as metagenomics, can be used to yield more information about phages in BWT samples but have their own limitations. The diversity of microbial communities is still poorly described. Therefore, many metagenomic analyses of the virome from BWT result in more than 95% of genome fragments having no homology with genomes deposited on databases (Tamaki et al., 2012; Petrovich et al., 2019). This leaves most of the phage community unidentified. This issue is further accentuated by the lack of genetic markers and the inability to design primers to establish an accurate phage identification and phylogeny (Angly et al., 2005; Gilbert and Dupont, 2011). The extrapolation of the outcomes from both approaches to phages needs to be carefully assessed (Handelsman, 2004; Angly et al., 2006; Hayes et al., 2017). Culture-independent methods are mostly developed and applied for the analysis of double-stranded DNA. Phages with single-stranded DNA and RNA might be excluded in such analysis and it is necessary to adjust the bioinformatics tools for much smaller and simpler genomes (Gilbert and Dupont, 2011; Ogilvie and Jones, 2015). Automatic annotation programs occasionally display false negatives corresponding to undetected genes, false positives for non-coding open reading frames identified as coding, and incorrect start codons assigned (Salisbury and Tsourkas, 2019). These lapses accumulate over time as the genome annotation for new phages relies on previous annotations. Nonetheless, research on phage ecology in BWT is still very limited and with scope to apply well-described culture-based and -independent methods.

When it comes to understanding the dynamics between phages and bacterial populations, the complex and variable nature of the microbial communities, wastewater influent, and operation of BWT extend to the phage-host dynamics. There are three major models to describe the ecology of phage-host systems: (1) the kill-the-winner model characterized by a boom-and-bust cycles between the phage and respective host abundances (Thingstad et al., 2008; Ogilvie and Jones, 2017); (2) the arms-race model referring to the co-evolution of phage and host, in which the bacterial host develops mechanisms to resist phage infection, which are continually countered by reciprocal adaptation of infecting phage (Thingstad, 2000; Hall et al., 2011); and (3) the fluctuating-selection model, where a balance in increase and decrease in populations is observed without eradication of phage or host, resulting in an established phage community and ensuring bacterial diversity (Mirzaei and Maurice, 2017). Although kill-the-winner is suggested to be the dominant model in BWT (Shapiro et al., 2010; Brown et al., 2019), limited data on phage identity and abundance over time compromise the complete description of dynamics between phages and hosts (Rohwer and Thurber, 2009; Shapiro et al., 2010). In full-scale systems treating continuous and variable influent streams and subject to seasonal changes, the impact of phage infection might be mitigated by a more diverse and robust microbial culture, while also conditioned by operation and abiotic factors (Shade et al., 2012). It is likely that all models are observed in BWT, with kill-the-winner being suggested as dominant, but the population dynamics remain to be fully described. Literature offers speculations regarding the prevailing models in BWT, without, however, providing authoritative supporting evidence (Rodriguez-Brito et al., 2010; Brown et al., 2019). In addition, it is to note that prophage inducers, such as chemicals and stress conditions, are present by default in BWT systems (Muniesa et al., 2011) – but their efficiency as prophage inducers in a full-scale system was never explored.

### Opportunities and Potential Application of Phages in BWT

Expanding research to investigate different BWT technologies and configurations is a chance to better understand the correlation between phages community, microbial community, and process. Consequently, it can yield crucial information to rethink the design and operation of BWT, and endorse biotechnological applications of phages. (Hatfull, 2008; Hayes et al., 2017).

High viral and microbial diversity of BWT constitute a valuable resource of useful – yet unknown – functional genes and organisms for biotechnological applications (Tamaki et al., 2012). Phage-mediated biocontrol, for example, for bulking and foam reduction, can be specific to the detrimental organisms and a more sustainable and low cost alternative to the use of chemical defoamers that are sometimes necessary in BWT tanks. Application of polyvalent phages can be useful as it can target multiple bacteria associated with an undesired phenomenon (Khairnar et al., 2014, 2016; Liu et al., 2015). Alternatively, a mixture of phages can target multiple detrimental bacteria or attach to different external receptors of the bacterial host (Oechslin, 2018). However, as mentioned in “Use of Specifically Isolated Phages for Bacterial Population Control,” the application of phages as biocontrol agents in wastewater treatment processes has not been investigated at larger or full-scale systems. Besides the replication of the phage to high volumes and to an adequate concentration for an effective population control, the formulation of the bioagent needs to be considered to ensure phage activity and effectiveness (Malik et al., 2017). For instance, phages can be lyophilized and added as a powder if a change in volume could negatively impact treatment performance. Furthermore, when developing a phage-mediated biocontrol, it is necessary to consider the possible rapid development of phage-resistant mutants, which can ultimately deteriorate the phage application (Labrie et al., 2010; Oechslin, 2018). Besides the scope to further investigate and develop the examples provided in “Use of Specifically Isolated Phages for Bacterial Population Control” into feasible applications, it can be interesting to exploit phages that infect bacteria not related to foam or bulking phenomena, but to functional aspects, such as carbon conversion or nutrient removal.

Phage-mediated control can also be adapted as a microbial ecology tool to study the function of a key population in BWT. Applying phages to infect and decrease the abundance of functional bacteria and assessing how system performance changes can yield information about the role of the decimated bacteria (Haruta et al., 2009). This can be a valuable strategy to correlate structure and function of microbial communities and help disclose the paradigm of microbial ecology of linking bacteria identity to function. However, this perspective diverges from the main directions of current research on phages in BWT, usually focused on tracking pathogenic bacteria and pollution (Bayat et al., 2021; Ji et al., 2021).

Phages could also be used as monitoring tools and performance indicators. This is a similar approach to the use of phages as pathogens indicators (McMinn et al., 2018), but rather than being detected to assess pollution levels or decay of enteric viruses, they could be used as indications of key functional bacteria of BWT systems. The detection and identification of particular phages can provide information about the health and stability of the bacterial community and help predict disturbances based on population dynamics of phage-host systems (Aw et al., 2014; Stefanakis et al., 2019). Since phages are more resistant than, for instance, enteroviruses (Mocé-Llivina et al., 2003), and considering the estimation that they can persist for up to 30days in the system (Tamaki et al., 2012), phages have the potential to be used as effluent quality indicators (Silverman et al., 2013; Yahya et al., 2015; Boehm, 2019). Further correlation of a phage ecogenomic fingerprint with treatment configuration and operation can evolve into a tool to monitor and control BWT in a quicker and more reliable way, again deviating from a health-related assessment toward a process and engineering perspective.

### Directions for Future Research

Culture-based and -independent methods complement each other and should be carried out in parallel. Metagenomics and molecular methods, increasingly accessible, should be more widely explored and applied in BWT microbial ecology research. There is an opportunity to sequence all phages isolated from BWT systems, as only a few had their genome sequenced. Increasing data available in public repositories can mitigate the misrepresentation of the BWT virome in genome databases. New genetic sequences and respective annotations will continuously build a landscape of phages found in BWT, similar to existing metagenomic databases specific for viruses in fresh (Roux et al., 2012) and sea water (Hurwitz and Sullivan, 2013). Metagenomic data can also be used to investigate patterns or clusters of organisms that are concurrent and thus help correlate bacteria identity with a specific function in the system (Otawa et al., 2007; Ogilvie et al., 2018). Other culture-independent methods, such as proteomics and novel techniques to study phage-host range, can be applied to BWT viromes and yield additional information on activity and life cycle of phages. Future research should also focus on the development of new strategies to counter host specificity and development host cell resistance as these highly compromise the understanding of the interaction between phages and bacteria and potential biotechnological applications.

Efforts toward developing fundamental knowledge on identity, diversity, and phage-host infection dynamics will support and expand the application of phages for monitoring and control in BWT. All the reported examples of phages used as biological control agents are from lab-scale systems and most of them with highly enriched microbial communities. Future experiments should investigate the applicability of such phages to microbial communities of full-scale systems that generally have higher microbial diversity due to more complex composition and variability of the influent and operating conditions. Such approaches also help mitigate biases associated with lab-acclimatized systems and provide a more reliable hypothesis for phage-host dynamics in real, full-scale systems.

Time series studies in full-scale BWT systems can complement the investigations on the dynamics between the viral and bacterial populations on both full- and lab-scale systems. Such studies also provide data for a more in-depth analysis of the impact that influent, operation, and environmental conditions can have on phage abundance and activity. Thereby, continuous changes and microbial interactions that cannot always be mimicked in pure cultures or lab-scale reactors are considered. A more comprehensive sampling of full-scale BWT systems is also encouraged. It has been suggested that bacterial strains can migrate and disperse through different systems but need to adapt to the local bacteriophage population (Kunin et al., 2008). Sampling different stages of the treatment process or points of the treatment tank increase the chance of getting a representative landscape of phage population in BWT and can yield information about the fate and migration of phages in the system (Otawa et al., 2007).

Another gap barely addressed is the functional influence of phages in BWT. Such impact can lead do a decrease in the abundance of useful microorganisms, impairing process performance, or be favorable to the microbial communities, or be favorable to the microbial communities, enhancing the resilience and stability of the culture or drive diversity and evolution. The effects of phage activity might be even more complex with different variations of life cycles taking place and constant co-evolution of phages and hosts. The virus-to-microbe ratio can be a good parameter to infer about lysogeny in the system, with low ratios usually being found in systems with high numbers of microbial cells and can indicate low propagation of phages and prevalence of lysogeny (Knowles et al., 2016). This parameter has not yet been explored for BWT systems. Statistical analysis and modeling of data collected from extensive sampling, over longer periods of time, can yield novel insights on the evolution of BWT viral and bacterial communities and the interactions shaping the structure and function of the system.

## Concluding Remarks

Although phages are active members of microbial communities in BWT, very little is known about their identity and activity, with studies often overlooking their impact on treatment performance. The role of phages is a missing but integral piece for fully understanding microbial ecology of BWT but also to improve and monitor wastewater treatment and to design innovative biotechnological solutions. This review provides routes for expanding the research of phages in BWT, including the most suitable methodologies and specific questions that need to be addressed. A deeper knowledge of phage ecology is a stepping stone in fully understanding the underlying microbial communities in BWT and is required to advance operation of full-scale systems.

## Author Contributions

The submitted review results from a project supervised by AL and BJ. AL, VR, BJ, and SB conceptualized the structure and content of the manuscript. VR did the literature research and wrote the first draft of the manuscript. All authors reviewed the manuscript, provided comments, and complemented the reference list. BJ contributed more significantly to “Introduction” and “Methods to Study Phages in BWT Systems,” while SB, JW, and AL to “Phages Identified in BWT Processes” and “Challenges and Opportunities for Studying Phages in BWT Systems.” All authors contributed to the iterative revision and edition process and approved the submitted version.

## Funding

This work was supported by the Engineering and Physical Sciences Research Council (grant number EP/L016354/1).

## Conflict of Interest

Author Simon Bengtsson was employed by company Promiko AB.

The remaining authors declare that the research was conducted in the absence of any commercial or financial relationships that could be construed as a potential conflict of interest.

## Publisher’s Note

All claims expressed in this article are solely those of the authors and do not necessarily represent those of their affiliated organizations, or those of the publisher, the editors and the reviewers. Any product that may be evaluated in this article, or claim that may be made by its manufacturer, is not guaranteed or endorsed by the publisher.
